# Exciton-plasmon coupling in tBLG-Au nanodisks for ultrasensitive miRNA sensing

**DOI:** 10.1093/nsr/nwaf479

**Published:** 2025-11-04

**Authors:** Huiling Lin, Zhi Chen, Changyu Yoon, Yubin Lee, Seokjin Hong, Jiwoo Seo, Xiangjiang Wang, Min-Goo Lee, Jong Seung Kim

**Affiliations:** The Affiliated Qingyuan Hospital (Qingyuan People’s Hospital), Guangzhou Medical University, China; Department of Chemistry, Korea University, Republic of Korea; Department of Chemistry, Korea University, Republic of Korea; Department of Chemistry, Korea University, Republic of Korea; Department of Chemistry, Korea University, Republic of Korea; Department of Chemistry, Korea University, Republic of Korea; The Affiliated Qingyuan Hospital (Qingyuan People’s Hospital), Guangzhou Medical University, China; Department of Chemistry, Korea University, Republic of Korea; Department of Chemistry, Korea University, Republic of Korea

Genetic detection technology enables accurate identification of nucleic acid sequences or variations and has been widely applied in life sciences, food safety, environmental monitoring, and clinical diagnosis. However, traditional genetic detection has significant limitations. First, it struggles with detecting low-abundance targets: clinically relevant targets such as circulating tumor DNA (ctDNA) and nucleic acids from low-load pathogenic bacteria are vulnerable to interference from sample matrices. Even with PCR amplification, false-negative results occur, and multiplex target detection suffers from probe cross-reactivity, leading to misdiagnosis or missed diagnosis [1]. Second, it has poor process adaptability, relying on professional operation and large-scale equipment, with strict requirements for environmental conditions and sample transportation, making it difficult to deploy in non-laboratory settings such as primary healthcare institutions and field locations. While traditional surface plasmon resonance (SPR)-based sensors enable label-free real-time detection [2], they are limited by pure optical signal transduction, making sub-femtomolar detection impossible and failing to meet practical demands for ultra-sensitive rapid diagnosis. Thus, there is an urgent need for rapid genetic detection technology with high stability, sensitivity, and specificity to enhance public health security management and improve patient prognosis. This work also follows the research trajectory established by Zhang and co-workers, whose 2022 study demonstrated a CRISPR-powered plasmonic sensing platform capable of precise variant discrimination with high sensitivity [3]. That pioneering work laid an important conceptual foundation for integrating programmable nucleic acid recognition with optical transduction, which is now further advanced and expanded in the present study.

**Figure 1.**
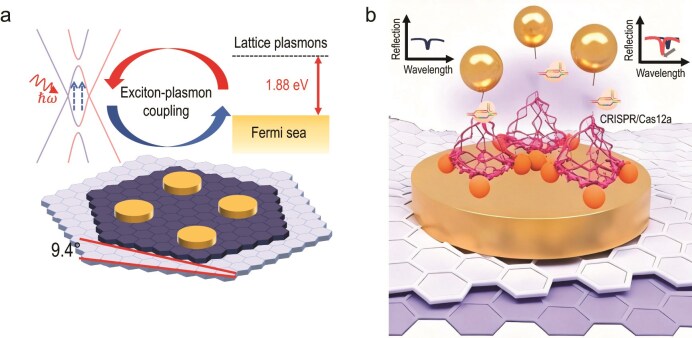
Mechanism of action of the biosensor based on twisted bilayer graphene (tBLG). (a) Illustration of the principle of exciton-plasmon coupling. (b) Biosensing platform architecture: reflection spectra show optical signal changes pre- and post-sensing. Adapted with permission from Ref. [6].

Among emerging materials for high-performance biosensing, twisted bilayer graphene (tBLG) shows excellent angle-tunable optoelectronic properties [4], but high photoresponse in tBLG-based sensors has been limited by reliance on intense illumination—a core bottleneck restricting its application [5]. To address this issue, Zhang and colleagues [6] proposed a groundbreaking approach in their latest study (Fig. 1). They precisely

tuned the van Hove singularity (VHS) absorption spectrum of 9.4° tBLG to align perfectly with gold nanodisks’ surface plasmon resonance under low power (60 μW) and achieved nanoscale positioning of gold nanoparticles (AuNPs) via DNA origami. This spectral alignment maximizes the joint density of states and exciton generation efficiency, thereby strengthening exciton–plasmon coupling and enhancing photocurrent output. This not only boosted photocurrent by 7-fold compared to pristine tBLG (overcoming intense illumination dependence) but also integrated CRISPR-Cas12a’s high-specificity trans-cleavage with photoelectric transduction to build a closed-loop sensing system (‘AuNP-mediated dielectric regulation-photocurrent conversion’), avoiding probe entanglement and non-functional occupation. Their sensor achieved multiple breakthroughs: 44.63 aM sub-femtomolar nucleic acid detection (without external amplification, a 4-order-of-magnitude sensitivity gain over traditional sensors), 14.64 mA/W photoresponsivity for the gold nanodisk/tBLG heterojunction (6.27-fold versus pristine tBLG), 27.51% external quantum efficiency, 1-hour detection time, and high stability after 20-day incubation in PBS/whole blood—laying a critical foundation for tBLG’s application in high-sensitivity, low-power biosensing that resists complex environments.

Clinically, the technology matches qPCR for miRNA-21 detection in plasma (with better variability), uses CRISPR-Cas12a for single-base resolution (reducing misdiagnosis), and extends to multi-biomarker detection via DNA origami. Such high specificity and sensitivity are particularly valuable for early-stage cancer diagnostics, minimal residual disease monitoring, and precise detection of rare point mutations, where conventional approaches often fail. Density functional perturbation theory calculation confirms that tBLG’s twist angle regulates dielectric constant (9.4° has higher in-plane value), enhancing photoelectric response and revealing the ‘twist angle-dielectric-photoelectric’ relationship. This approach integrates technologies to overcome traditional limitations, building a universal ultra-sensitive biosensing framework.

In summary, Zhang’s group successfully combines technological breakthroughs with clinical applications. By pioneering ‘moiré material engineering + programmable nanointerface’, they break traditional sensing boundaries, enabling diverse biomarker detection and marking a significant milestone for precision medicine.
